# The spectrum of non- neoplastic skin lesions in Ibadan, Nigeria: a histopathologic study

**DOI:** 10.11604/pamj.2016.23.221.9372

**Published:** 2016-04-22

**Authors:** Gabriel Olabiyi Ogun, Obumneme Emeka Okoro

**Affiliations:** 1 Department of Pathology, University of Ibadan/ University College Hospital, Ibadan, Nigeria; 2 Dermatology unit, Department of Medicine, University of Ibadan/ University College Hospital, Ibadan, Nigeria

**Keywords:** Skin lesions, histopathology, non-neoplastic, Ibadan

## Abstract

**Introduction:**

Non-neoplastic skin lesions constitute the majority of skin diseases. There is a paucity of histopathology studies of non-neoplastic skin diseases in Nigeria and the West Africa sub-region in general. This is because the dermato-pathology sub-specialty is poorly developed. Therefore, the main aim of this study is to determine the spectrum of histologically diagnosed non-neoplastic skin lesions in Ibadan, Nigeria.

**Methods:**

This is a retrospective study. All non-neoplastic skin lesions diagnosed in the Department of Pathology, University College Hospital, Ibadan over a five year period. (January 2006 to December 2010) was reviewed. The lesions were classified into eight groups according to the International Classification of Diseases (ICD)-10 of skin and subcutaneous disorders. The main classes include Dermatitis/Eczema, Papulosquamous disorders, Infectious disorders, Connective tissue diseases, Bullous disorders, Naevi/Developmental lesions, Granulomatous lesions, keratinizing disorders and other categories/Miscellaneous group.

**Results:**

A total of 209 non-neoplastic skin lesions comprised 1.3% of all surgical pathology specimen received within the study period. The modal age group was 20-29. The Dermatitis/Eczema group has the highest frequency of 87 cases representing 41.6% of cases, papulosquamous disorders 39 (18.7%), infectious disorders 37 (17.7%), bullous disorders 11 (5.3%) and connective tissue disorder 9 (4.3%). Chronic non-specific dermatitis was the commonest specific diagnosis comprising 60 cases (28.7%) of all the skin diseases. The other common specific skin lesions were lichen planus/lichenoid dermatitis 27(12.9% of 209 cases), verruca vulgaris 25 (12% of 209 cases).

**Conclusion:**

The number of histologically diagnosed non-neoplastic skin lesions is relatively small. There is a very wide spectrum of non-neoplastic skin lesions diagnosed within this period. There is a need for a specific diagnosis considering the high frequency of chronic non-specific dermatitis.

## Introduction

There are at least 2000 different skin diseases in the field of dermatology which cuts across all age groups [1]. Non-neoplastic skin lesions form the majority of the morbidity from skin diseases [2–6]. Although some of these lesions are easy to diagnose clinically, a substantial number will present a great challenge in diagnosis and management. Such cases are required to be properly diagnosed to enable appropriate management. Often, a good clinical history, adequate and optimal skin biopsy with a good histopathologic examination will likely leady to a satisfactory diagnosis. The field of dermato-pathology is poorly developed in Nigeria, in the West Africa sub-region and sub-Saharan Africa [7]. Therefore, in most circumstances general anatomic pathologists have to take up the burden of reporting skin biopsies, usually without the attendant intricate knowledge needed for this. After a skin biopsy for a patient, the appropriate diagnosis may require a form of interaction between the dermatologist and the anatomic pathologist for difficult and challenging cases. This may be by formal or informal interactions or meetings, serving as the platform to give a good clinico-histopathologic correlation and ultimately a final diagnosis for such cases. Histological examination may not be required for definitive diagnosis of majority of non-neoplastic skin lesions. However, the knowledge of the spectrum of histologically diagnosed non-neoplastic skin lesions can give an insight into the relative frequency of these lesions. This will likely help dermatologists to have an idea of which lesions, they will likely continue to biopsy especially when there is a consistent poor clinic-pathologic correlation. The aim of this study was to review all cases of non-neoplastic skin biopsies diagnosed over a period of 5 years (January 2006 to December 2010) at the department of Pathology, University College Hospital, Ibadan, Nigeria.

## Methods

This was a retrospective study. All cases of skin biopsy with a non-neoplastic diagnosis were retrieved from the records and database of Department of Pathology, University College Hospital (UCH), Ibadan, Nigeria, to cover a 5-year period, January 2006 to December 2010. The cases must have a final diagnosis to be included in the study. All the patients were Nigerians. The patients’ basic clinical data including age, gender were noted. The lesions were classified into groups according to the International Classification of Diseases (ICD)-10 for disorders of skin and subcutaneous tissue for ease of analysis and comparision with other studies. The classes include Dermatitis/Eczema group, Papulosquamous lesions, Infectious disorders, Connective tissue diseases, Bullous disorders, Naevi/Developmental lesions, Granulomatous lesions, keratinizing disorders and other categories/Miscellaneous group. All the cases were reviewed by **GOO** to corroborate the diagnosis and stratified them into the appropriate class. The data was entered and analysed using SPSS 16.0 statistical software. Simple descriptive analysis was used to analyse the parameters like age and gender. The frequency of other variables like histological diagnosis, group of skin diseases were expressed in percentages. This study was conducted in compliance with the guidelines of the Helsinki declaration on biomedical research in human subjects. Confidentiality of the identity of the patients and personal health information was maintained. This study was limited by its retrospective design and being a histopathologic study.

## Results

**Figure 1:**
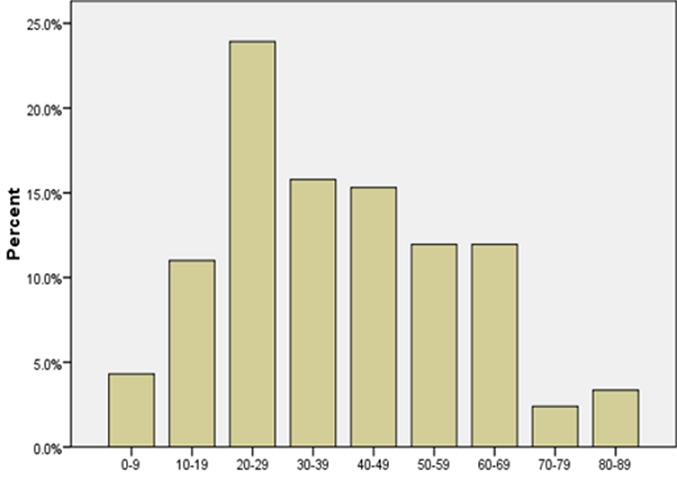
Age group of patients with non-neoplastic skin diseases from this study

A total of 209 cases fulfilled the inclusion criteria for the study. This represented 1.3% of all surgical biopsy specimens received in the Department of Pathology, University College Hospital, Ibadan, Nigeria over the study period. One hundred and five cases (50.2%) were females while 104 (49.8%) were males giving a male: female ratio of 1. The mean age of all the patients was 38.8years while the median age was 37 years. The mean age for males and female was 41.5years (SD 19.6) and 36.3 years (SD 18.2) respectively. The modal age group was 20-29 years (Figure 1) representing 23.9% of the total number of cases in this study. Table 1 show all the disease categories/ stratification in this study based on the ICD-10 of the disorders of skin and subcutaneous tissue. In all, 8 categories/disease groups had 3 or more cases represented in each group and are highlighted as follow.

**Table 1:** The frequency of group of diseases in this study based on ICD-10

ICD-10 Group	Number of cases	%
Dermatitis/Eczema	87	41.6
Papulosquamous disorders	39	18.7
Infectious disorders	37	17.7
Bullous disorders	11	5.3
Connective tissue disorders	9	4.3
Naevi/Developmental lesions	8	3.8
Granulomatous disorders	7	3.3
Keratinizing disorders	3	1.4
Others lesions	8	3.8
**Total**	**209**	**100.0**

**Dermatitis/eczema group**: This group has the highest frequency of 87 cases representing 41.6% of the total number of cases in this study (Table 1). Chronic non-specific dermatitis accounts for 68 cases of the overall total of 209 cases and accounts for 78% of the 87 cases in the dermatitis/eczema group. There were 15 cases of eczematous/spongiotic dermatitis and a single case of lichen simplex chronicus, acute necrotizing dermatitis, chronic necrotizing dermatitis and exfoliative dermatitis.

**Papulosquamous disorders**: This group comprises of 39 (18.7%) of the 209 skin diseases. Lichenoid dermatitis has the highest frequency of 19 (48.7%) of the 39 cases in this group (Table 2).

**Table 2:** Papulosquamous disorders frequency in this study based on ICD-10

Specific group	No	%
Lichenoid dermatitis	19	48.7
Lichen planus	8	20.5
Psoriasiform dermatitis	5	12.8
Psoriasis vulgaris	2	5.1
Lichen nitidus	2	5.1
Pityriasis rosea	1	2.6
Pityriasis lichenoides chronica	2	5.1
**Total**	**39**	**100.0**

**Infectious diseases**: Infectious diseases comprises 37 (17.7%) of the 209 skin diseases. Viral infections has the highest frequency of 32 (86.5%) out of the 37 infectious diseases, Table 3.

**Table 3:** Infectious disease category with specific disease entity

Infectious disease groups and with specific disease for each group	No	%
Viral (Verruca Vulgaris = 25; Molluscum contagiosus= 7)	32	86.5
Bacterial (Carbuncle = 1; Lepromatous Leprosy = 1)	2	5.4
Fungal (Chromomycosis = 1; Onychomycosis = 1)	2	5.4
Protozoa (Cutaneous Leishmaniasis = 1)	1	2.7
**Total**	**37**	**100.0**

**Nevi/developmental disorders**: There were 8 cases in this group representing 3.8% of the 209 cases in this study. There were 5 cases of melanocytic nevus, 2 cases of linear epidermal nevus and a single case of vascular nevi.

**Bullous disorders:** Comprises 11(5.3%) of the 209 skin diseases. Bullous pemphigoid comprises 5 (45.5%) of the 11 bullous disorders. There were 2 cases of pemphigus foliaceus, and a single case each of bullous impetigo, dermatitis herpetiformis, pemphigus vulgaris and epidermolysis bullosa.

**Connective tissue diseases**: DLE comprises 6 (75%) of the connective tissue diseases but only 2.9% of the 209 skin lesions in this study. There was a single case each of Systemic Lupus Erythematosis, scleroderma and small vessel vasculitis

**Granulomatous disorders**: Comprises 7 (3.3%) of the 209 skin diseases. There were 4 cases of cutaneous sarcoidosis, two cases of granulomatous dermatitis where the aetiology of the granulomas could not be ascertained and a single case of necrobiopsis.

**Keratinizing disorders**: Comprised of two cases of lamellar ichthyosis of sisters in the same family and a single case of keratosis follicularis.

**Others categories/Miscellaneous group**: The other cases identified in the study include 5 cases of calcinosis cutis and a single case of erythema dyschronicum, erythema multiforme and vitiligo.

## Discussion

This present study had 209 cases that were analysed. This histopathologic study has smaller number of cases when compared to clinically based studies of non neoplastic skin lesions [2–7]. Purely clinical based studies of non-neoplastic skin lesions, obviously, report much higher number of cases for similar study periods as is the case in a previous study from our centre [2]. This clearly illustrates that most skin lesions do not get biopsied for histopathologic assesement as majority of dermatologic cases can be treated and managed clinically. There are no readily available studies or cohort that we can compare our findings with because most studies of non- neoplastic skin lesions are usually not histopathology based. The mean age of the all the patients we studied was 38 years, with the peak age group been the 20-29 age bracket. This group comprises mainly young adult who are very active and probably exploring various ways of improving their skin outlook and this may have predisposed them to skin lesions. Moreover, they are likely to be worried about their skin for cosmetic reasons. Therefore, they are more likely to visit the dermatologists and apt to have the lesions biopsied for histopathologic assessment. The Dermatitis/Eczema group constitutes the largest group of diseases in this current study. This may be a correlation to the rising frequency of eczematous lesions in clinical based studies of skin lesions from our environment [2, 3]. Nevertheless, the high frequency of non-specific dermatitis in the dermatitis/eczema group is attributable to the common reaction pattern of many non-neoplastic skin lesions. The frequency of Papulosquamous lesions was relatively high in this study with lichenoid dermatitis and lichen planus being very common. The persistent irritating and poor cosmetic effects of these lesions may explain the willingness of this group of patients having biopsy for histopathology to establish a specific diagnosis. Among the infectious diseases group, viral infections had the highest frequency with verrucae vulgaris being the commonest. The excision biopsy of such lesion, apart from helping to arrive at a definitive histopathologic diagnosis, is also therapeutic and serves as good cosmetic outcome for the patient. Superficial fungal infections which are the commonest causes of skin infections based on clinical studies are not prominent in our current study because these lesions do not usually require biopsy for diagnosis and therapy. Connective tissue diseases constitute 3.8% of the skin lesions. This is similar to the frequency of connective tissue disorders reported in clinical based studies in Nigeria [2, 3]. The relatively higher frequency of discoid lupus erythematosus observed in this study was also reported in those clinical based studies. The frequency of bullous disorders was 5.3%. This is high compared with hospital and community based studies in our environment. Bullous pemphigoid constitute 45.5% of bullous lesions. This was rarely reported in clinical based studies. Many of the patients with bullous skin lesions are admitted through the emergency unit; therefore they were rarely reported in the clinical based studies. Naevi/developmental disorders comprise 3.8% of the skin lesions. This is similar to its frequency observed in a community based studies among school children in Ibadan [6]. These lesions are relatively rare in black Africans when compared to Caucasians [2, 6]. Granulomatous disorders comprise 3.3% of the skin lesions. This frequency is also relatively high compared with frequency of granulomatous diseases in clinic based studies. Cutaneous sarcoidosis has the highest frequency. This was rare in the clinic based study done in Ibadan. Usually this lesion maybe diagnosed clinically, however definitive therapy cannot be initiated unless a histopathologic diagnosis is known hence the need for a biopsy. This study illustrates that granulomatous lesions and connective tissue disorders and some other lesions that have specific therapy, for example cutaneous sarcoidosis, where the use of steroids is considered as part of the therapeutic management, will usually have a biopsy performed no matter how confident the clinical diagnosis is. This is based on the fact that the patient might have to use the therapy for prolonged period of time and with the attendant possible complications of therapy. The two cases of Lamellar ichthyosis in the Keratinizing disorders group occurred in sisters of the same family and is already documented in literature [8]. A genetic predisposition is presumed because it is clustered in and run in this family. This study was limited by its retrospective design and being a histopathologic study, but gives an insight into the limitations and constrain in clinical management when histopathologic asssesement of a lesion is not performed especially for granulomatous and connective tissue disorders.

## Conclusion

Non-neoplastic skin lesions constitute a small percentage of the biopsies done in our practice setting. There was a very wide spectrum of skin lesions observed among patients thus the need to confirm diagnosis by biopsy to ensure proper management. The high frequency of non-specific dermatitis as a final diagnosis may reflect the common reaction and non-specific pattern of many non-neoplastic skin lesions. The frequencies of specific bullous and granulomatous disorders were higher when compared to frequency from clinically based studies which indicate the limitation of clinical assessment in giving a definitive diagnosis in such lesions. There is need for a careful and detailed evaluation of skin lesions by both clinicians and pathologists to reduce the frequency of these non-specific diagnoses.

### What is known about this topic

- Non-neoplastic skin lesions are diverse and many cases have similar presentation clinically;
- Majority of non-neoplastic skin lesions are not biopsied, hence not diagnosed by histopathology.

### What this study adds

- Histopathologic diagnoses serve as the gold standard for lesions that require long term therapy;
- Non-specific dermatitis as a final histopathologic diagnosis may reflect the common reaction and non-specific pattern of many non-neoplastic skin lesions.
